# The role of genetic polymorphisms of interleukin-1 (IL-1R1 and IL-1RN) in primary knee osteoarthritis in Indonesia

**DOI:** 10.1038/s41598-023-34824-2

**Published:** 2023-05-17

**Authors:** Nicolaas C. Budhiparama, Imelda Lumban-Gaol, Herawati Sudoyo, Rahadyan Magetsari, Tri Wibawa

**Affiliations:** 1 Department of Orthopaedic and Traumatology, Faculty of Medicine, Unversitas Airlangga, Surabaya, Indonesia; 2 Nicolaas Institute of Constructive Orthopaedics Research and Education Foundation for Arthroplaty and Sports Medicine, Jakarta, Indonesia; 3 Eijkman Institute for Molecular Biology, Jakarta, Indonesia; 4 Department of Orthopaedic and Traumatology, Faculty of Medicine, Public Health, and Nursing, Universitas Gadjah Mada, Yogyakarta, Indonesia; 5 Department of Microbiology, Faculty of Medicine, Public Health, and Nursing, Universitas Gadjah Mada, Yogyakarta, Indonesia; 6 Department of Orthopaedics, Leiden University Medical Centre, Leiden, The Netherlands

**Keywords:** Genetics, Genetic association study, Genome-wide association studies

## Abstract

This study aimed to evaluate the association of SNPs of the IL-1 family with the clinical severity of knee OA. This case‒control study was performed among 100 healthy knees and 130 osteoarthritis (OA) knees of people aged ≥ 50 years with a BMI ≥ 25 kg/m^2^. The possible correlations among clinical findings, radiographic evaluations, serum levels of IL-1R1 and IL-1Ra, and genotype analyses were evaluated. Three SNPs of IL-1R1, rs871659, rs3771202, and rs3917238, were associated with primary knee OA. Females with IL-1R1 SNP rs871659 allele A had a higher prevalence of primary knee OA. No correlation was found between SNPs of IL-1R1 and IL-1RN and clinical or radiologic severity or serum concentrations of IL-1R1 and IL-1Ra (*p* > 0.05). BMI and IL-1R1 rs3917238 genotype C/C were correlated with moderate-severe VAS scores. A correlation was also found between the EQ-5D-3L self-care dimension and obesity and between the EQ-5D-3L pain and usual activity dimensions and age ≥ 60 and obesity (*p* < 0.05). Radiologic severity was only associated with age ≥ 60 years (*p* < 0.05). We found the IL-1R1 SNPs rs871659, rs3771202, and rs3917238 to be predisposing factors for primary knee osteoarthritis. The clinical findings, radiographic severity, and serum concentrations of IL-1R1 and IL-1Ra were not correlated with these gene polymorphisms.

## Introduction

Osteoarthritis (OA) is the most common form of arthritis^1^ and the most prevalent chronic joint disease^2^. OA is a multifactorial inflammatory and degenerative joint disorder^3^. In the inflammatory mechanism, the proinflammatory cytokine that plays an important role is IL-1β, produced by the IL-1 gene. IL-1β binds to the IL-1 receptor (IL-1R1), competes with IL-1Ra (IL-1 receptor antagonist protein), and induces a proinflammatory reaction^4^.

Several groups of investigators have performed genome-wide sequencing, which showed that the most consistent linkage was reported for the interleukin-1 (IL-1) gene cluster, including single-nucleotide polymorphisms (SNPs) in IL-1β, IL-1R1, and the interleukin-1 receptor antagonist gene (IL-1RN)^5^. Several IL-1 SNPs have been proven to be associated with knee OA in Caucasians, but different IL-1 SNPs have been associated with knee OA in Asians. These results were conflicting^6–9^. Therefore, in this study, we used whole genome sequencing, which is a recent advance in genomic studies, to determine the order of nucleotides in entire genomes or targeted regions and discover new SNPs that correlate with primary knee OA and clinical findings. We evaluated not only the IL-1 SNPs that have been proven (IL-1RN gene variants rs419598, rs9005, and rs315943 and IL-1R1 rs2287047) but also new SNPs that might correlate with knee OA. These new SNPs can be used in genomic data worldwide. To date, there has been no research focusing on the entire genome or regions of IL-1 gene cluster polymorphisms and their association with knee OA.

The main purpose of this study was to investigate the IL-1RN gene variants rs419598, rs9005, and rs315943 and IL-1R1 rs2287047 and also whole genome of the IL-1 gene cluster polymorphism of IL-1β (IL-1R1 and IL-1RN) in primary knee osteoarthritis and its association with clinical findings, severity of knee osteoarthritis and corresponding biomarkers.

## Materials and methods

This was a case‒control study of primary knee osteoarthritis patients in Mongoloid who sought medical treatment at our orthopaedic outpatient clinic from October 2019 to August 2021. The control group was selected from patients’ relative and friends who has no knee complaint who fulfilled the inclusion and exclusion criteria. The inclusion criteria for controls were healthy knees, age over 50 years with a BMI over 25 kg/m^2^, no knee pain (VAS score = 0), no functional knee limitations (Knee Injury and Osteoarthritis Outcome Score (KOOS) = 100), and normal knee radiology. For the case group, the inclusion criteria were patient age over 50 years with a BMI over 25 kg/m^2^ who were diagnosed with primary knee osteoarthritis radiologically and had knee pain or functional limitations. All subjects in both groups agreed to follow all research procedures, including structured interview sessions and complete physical examinations by the principal investigator or appointed research assistant in this study. The exclusion criteria for the case and control group were patients with trauma or fracture around the knee, previous traumatic knee injury or any history of trauma, arthritis, ankylosing spondylitis, septic arthritis, other arthritis or any other systemic inflammatory or autoimmune disorder, and any cancer. Based on a sample size calculation with a power of 80%, β = 0.2 and α = 0.05 using binomial proportions, the sample for the control group was 100 patients, and the sample for the case group was 130 patients, who were divided into grade 2 with mild OA (65 patients) and grades 3–4 with moderate-severe OA (65 patients). We evaluated the clinical findings (using the VAS, EQ-5D-3L, and KOOS), radiological scores (Kellgren-Lawrence), laboratory findings (protein IL-1R1 and IL-1Ra) in plasma, and genetic polymorphisms of IL-1R1 and IL-1RN with 4 SNPs (IL-1R1_rs228704, IL-1RN_rs419598, IL-1RN_rs9005, and IL-1RN_rs315943) based on previous studies performed by Attur et al.^10^, Wu et al.^7^, and Ahmed et al.^8^. To screen other SNPs that might correlate with primary knee OA, we performed next-generation sequencing (NGS). This research was approved by the Ethics Review Committee of the Faculty of Medicine, Public Health and Nursing, Universitas Gadjah Mada.

### Blood samples

Blood samples were collected after informed consent was obtained from patients with normal knees and those with knee osteoarthritis diagnosed by radiograph examination. Patients and families were informed that samples would be taken to establish solid diagnoses and for research purposes. For DNA isolation (genotype analysis), we used EDTA tubes to keep whole blood, while for ELISA (phenotyping analysis), we used blood serum to exclude any inflammatory disease and cancer.

Serum IL-1Ra and IL-1R1 concentrations were measured using Quantikine^®^ ELISA Human IL-1Ra/IL-1F3 (R&D Systems, Inc., Minneapolis, USA, Cat: DRA00B, Lot: P296268), DuoSet^®^ ELISA Development System Human IL-1R1 (R&D Systems, Inc., Minneapolis, USA, Cat: DY269, Lot: P202606) and DuoSet^®^ Ancillary Reagent Kit 2 (Cat: DY008, Lot: P302483).

### Phenotyping analysis

To evaluate serum IL-1Ra, human IL-1Ra standard was reconstituted with distilled water to make a standard stock solution with a concentration of 20,000 pg/mL. The standard stock solution was then diluted at a 1:1 ratio with Calibrator Diluent RD5-33 (2X dilution per level) to achieve the following concentrations (Calibrator Diluent RD5-33 was also used as standard 0 pg/mL). A total of 100 µL of Assay Diluent RD15 was added to each well. Standards and samples were put into the wells according to the following procedure. Plates were incubated using Lovibond TC 135 S (Tintometer GmbH, Dortmund, Germany) for 2 h at room temperature (± 20 °C). The plates were washed with wash buffer 4 times using an automated washer, Biochrom Anthos Fluido 2 Microplate Washer (Biochrom Ltd., Cambridge, UK). A total of 200 µL of human IL-1Ra conjugate was added to each well. Plates were incubated for 2 h at room temperature (± 20 °C). The plates were washed with wash buffer 4 times using an automated washer. Substrate solution was prepared by mixing Color Reagent A and Color Reagent B at a ratio of 1:1. A total of 200 µL of substrate solution was added to each well. Plates were incubated for 30 min at room temperature. After the addition of substrate and incubation for 30 min, the liquid in the well appeared blue. A total of 50 µL of Stop Solution was added to each well. After the addition of Stop Solution, the liquid in the well changed colour from blue to yellow. Absorbance was measured with a microplate reader Bio-Rad model 680 (Bio-Rad Laboratories Inc., CA, USA) and Microplate Manager ver. 5.2.1 software (Bio-Rad Laboratories Inc., CA, USA) at a wavelength of 450 nm and wavelength correction at 540 nm. Standard curves were made based on optical density (OD) and standard concentrations of IL-1Ra. The concentration in the sample was determined based on the standard curve equation.

To evaluate serum IL-1R1, the capture antibody stock solution was diluted with PBS. A total of 100 µL of the diluted capture antibody was added to each well. The plate was covered with a plate sealer and then incubated overnight at room temperature (± 20 °C). The solution in the well was discarded, and then the plate was washed with wash buffer 3 times. A total of 300 µL of reagent diluent was added to each well (blocking stage). Plates were incubated for a minimum of 1 h at room temperature. The plates were washed with wash buffer 3 times. The plates were then ready to be used for adding samples. Human IL-1R1 standard was reconstituted with distilled water to make a standard stock solution with a concentration of 8000 pg/mL. The standard stock solution was then diluted at a 1:1 ratio (2X dilution) or 1:2 ratio (3X dilution) with Reagent Diluent to achieve the following concentrations (Reagent Diluent was also used as standard 0 pg/mL). Standards and samples were put into the wells in the following procedure. A total of 100 µL of standard 1 to standard 9 was added to wells A1 to D3. A total of 100 µL of the sample was added to wells E3 to H12. Plates were incubated for 2 h at room temperature (± 20 °C). The plates were washed with wash buffer 3 times using an automated washer. A total of 100 µL of detection antibody was added to each well. Plates were incubated for 2 h at room temperature. The plates were washed with wash buffer 3 times using an automated washer. A total of 100 µL of streptavidin-HRP was added to each well. Plates were incubated for 20 min at room temperature. The plates were washed with wash buffer 3 times using an automated washer. Substrate solution was prepared by mixing Color Reagent A and Color Reagent B at a ratio of 1:1. A total of 100 µL of substrate solution was added to each well. Plates were incubated for 20 min at room temperature. After the addition of substrate and incubation for 20 min, the liquid in the well appeared blue. A total of 50 µL of Stop Solution was added to each well. After the addition of Stop Solution, the liquid in well 152 changed colour from blue to yellow. Absorbance was measured with a microplate reader (Bio-Rad Laboratories Inc., CA, USA) and Microplate Manager ver. 5.2.1 software (Bio-Rad Laboratories Inc., CA, USA) at a wavelength of 450 nm and wavelength correction at 540 nm. Standard curves were made based on optical density (OD) and standard concentrations of IL-1R1 on the concentration in the sample was determined based on the standard curve equation.

### Genotyping analysis

All genotyping was performed in a clinical genetics laboratory (Eijkman Institute for Molecular Biology). For genotyping analysis, we first designed primers for the targeted sequences of the IL-1R1 and IL-1RN genes. DNA was extracted from blood samples using standard protocols. Before polymerase chain reaction (PCR), DNA was diluted to adjust concentrations to within a range compatible with multiplex PCR conditions. Then, we performed sequence amplification using PCR and next-generation sequencing (NGS), followed by analysing the data obtained. We used NGS to screen other polymorphisms that might correlate with primary knee OA. This protocol used the Nextera XT DNA Library Prep Kit (Illumina) according to the manufacturer’s instructions. Purified DNA from the same sample was pooled together. Pooled DNA was fragmented using an enzyme or sonicator and tagged with indices. All samples were amplified again using a thermal cycler to increase the amount of DNA in the library. Excess reagent was cleaned from the amplified library. The randomness of library amplification could lead to different amounts of amplified library for each sample. Therefore, it was necessary to normalize the libraries to even the amounts of each amplicon. All cleaned DNA libraries were pooled together for cluster generation. The index in each DNA sample acted as a unique marker to differentiate each sample for data analysis. We used GRCh38(hg38) as a reference for genotype analysis. The sequence of each DNA strand was read at the same time when new DNA strands were being synthesized in the flow cell. Cluster generation and sequencing were performed in a sequencer (Illumina MiSeq Next Generation Sequencer). DNA sequencing was analysed with bioinformatics. The primer sequences for this research are described in Table 1.

**Table 1:** Primers for SNPs in this research.

No	Variant rs	Forward primer	Reverse primer
1	IL-1RN 1	5’-GCA AGG TGT TCC GTC TCT T-3’	5’-GCC AAG GAC ATA GTC AAC AAT-3’
2	IL-1RN 2	5’-TGT ATG AAA GAT GGC TGT GC-3’	5’-CAA GTC CCC AGT CTT CTC AAA-3’
3	IL-1R1	5’-ATG TGT TCT TCC TTC CCC AGG-3’	5’-ACA GAG ACG AGT ATG AGA AAT GAC-3’

### Data analysis

Genotyping performance was assessed as the total genotyping success rate, genotype concordance rate between duplicates, and Hardy–Weinberg equilibrium (HWE). SNPs with minor allele frequencies < 0.01 were excluded. We analysed the data using crosstabulation with chi-square tests for nominal data. The independent t test or Mann‒Whitney U test was used for ratio/interval data, depending on the data distribution. We also evaluated the correlation of genetic polymorphisms with clinical findings using coefficient contingency for nominal data. Logistic regression analysis was used to examine the association between SNP variants and sex, age, BMI, clinical findings, radiologic severity, and laboratory findings. We used the smallest frequencies of alleles and genotypes as a reference, as we did not have any genotype mapping information for the Indonesian population. All statistical analyses were performed using IBM Statistical Package for the Social Sciences version 24.0 (SPSS Inc., Chicago, IL, USA).

### Ethical approval

The research protocol was approved by the Ethical Review Committee of the Faculty of Medicine, Public Health and Nursing, Universitas Gadjah Mada (Date: 30th Dec 2020, Number: 46); this study was performed in compliance with the 2013 Declaration of Helsinki.

## Results

The total number of patients in the control group was 100, with 72 (72%) females and 28 (28%) males and an average age of 57.35 ± 6.34 years. In the case group, there were 130 patients, divided into 65 patients in the mild subgroup and 65 patients in the moderate-severe subgroup. In the mild subgroup, 48 (73.85%) patients were female, and 17 (26.15%) were male, with an average age of 58.91 ± 6.85 years. The moderate-severe subgroup consisted of 51 females (78.46%) and 14 males (21.54%), with an average age of 62.00 ± 7.27 years, as shown in Table 2.

**Table 2:** Characteristics of the participants.

Variable	Control (Mean ± SD)	Mild OA (Mean ± SD)	Moderate-severe OA (Mean ± SD)	*p* value
Age	57.35 ± 6.34	58.91 ± 6.85	62.00 ± 7.27	0.000
Sex, N (%)				
Female	72 (72%)	48 (73.85%)	51 (78.46%)	0.566
Male	28 (28%)	17 (26.15)	14 (21.54%)	
BMI	26.03 ± 1.65	26.52 ± 2.44	27.51 ± 3.21	0.001
VAS score	1.42 ± 2.52	2.22 ± 2.20	4.08 ± 2.20	0.000
KOOS pain	94.47 ± 12.16	80.42 ± 22.02	50.14 ± 32.52	0.000
KOOS symptoms	69.96 ± 12.00	61.26 ± 16.41	48.10 ± 14.34	0.000
KOOS ADL	94.81 ± 13.31	81.72 ± 23.72	53.67 ± 32.20	0.000
KOOS sport	90.05 ± 21.11	73.69 ± 29.38	41.46 ± 32.43	0.000
KOOS QoL	87.94 ± 19.67	76.73 ± 21.89	44.53 ± 27.61	0.000
KOOS	84.59 ± 18.10	74.77 ± 20.47	46.85 ± 25.10	0.000
EQ-5D-3L mobility, N (%)				
No problems	95 (95%)	51 (78.46%)	23 (35.38%)	0.000
Some problems	5 (5%)	12 (18.46%)	39 (60%)	
Extreme problems	0	2 (3.08%)	3 (4.62%)	
EQ-5D-3L self-care, N (%)				
No problems	99 (99%)	62 (95.38%)	37 (56.92%)	0.000
Some problems	1 (1%)	3 (4.62%)	27 (41.54%)	
Extreme problems	0	0	1 (1.54%)	
EQ-5D-3L usual activity, N (%)				
No problems	95 (95%)	56 (86.15%)	30 (46.15%)	0.000
Some problems	5 (5%)	9 (13.85%)	34 (52.31%)	
Extreme problems	0	0	1 (1.54%)	
EQ-5D-3L pain, N (%)				
No problems	85 (85%)	32 (49.23%)	14 (21.54%)	0.000
Some problems	14 (14%)	31 (47.69%)	48 (73.84%)	
Extreme problems	1 (1%)	2 (3.08%)	3 (4.62%)	
EQ-5D-3L anxiety/depression, N (%)				
No problems	95 (95%)	58 (89.23%)	35 (53.85%)	0.000
Some problems	5 (5%)	6 (9.23%)	29 (44.61%)	
Extreme problems	0	1 (1.54%)	1 (1.54%)	
EQ-5D-3L VAS	83.23 ± 11.55	79.04 ± 13.06	73.04 ± 11.75	0.000
EQ-5D-3L index	0.856 ± 0.095	0.736 ± 0.208	0.542 ± 0.239	0.000
IL-1R1	37.51 ± 73.22	25.72 ± 39.86	24.62 ± 67.97	0.349
IL-1Ra	463.59 ± 197.00	470.29 ± 176.08	495.17 ± 214.36	0.590

We did not find any difference in the frequency of 4 SNPs (IL-1R1_rs228704, IL-1RN_rs419598, IL-1RN_rs9005, and IL-1RN_rs315943) that we targeted. However, we found another SNP that showed differences between groups (IL-1R1_rs871659 and IL-1R1_rs3771202) in terms of genotype (Table 3) or (IL-1R1_rs871659, IL-1R1_rs3771202, and IL-1R1_rs3917238) allele (Table 4). Therefore, the SNPs that we analysed further were IL-1R1_rs871659, IL-1R1_rs3771202, and IL-1R1_rs3917238. The gene variant rs871659 is located on chromosome 2:102,155,395 (GRCh38.p13), rs3771202 is located on chromosome 2:102,156,209 (GRCh38.p13) and rs3917238 is located on chromosome 2:102,156,623 (GRCh38.p13). All three gene variants are located in the IL-1R1 gene and are intron variants. In genotype analysis, we found that rs871659 and rs3771202 were associated with the incidence of mild OA compared to controls, and we found that rs871659 A/A and rs3771202 G/G had the highest prevalence among mild OA cases. In allele analysis, we found that rs871659 allele A, rs3771202 allele G, and rs3917238 allele T were associated with the incidence of mild OA compared to controls.

**Table 3:** Comparison of IL-1R1 and IL-1RN SNP genotypes between groups.

Variable (Genotype)		Group						*p* value (CS)	*p* value Control versus Mild	*p* value control versus severe	*p* value mild versus severe
		Control		Mild OA		Severe OA					
		n	%	n	%	n	%				
IL-1R1 rs2287047	G/G	40	40	16	24.6	22	33.8	0.315	0.282	0.700	0.700
	G/A	42	42	31	47.7	29	44.6				
	A/A	18	18	18	27.7	14	21.5				
IL-1RN rs419598	T/T	77	77	48	73.8	53	81.5	0.821	0.859	0.859	0.859
	T/C	22	22	16	24.6	11	16.9				
	C/C	1	1	1	1.5	1	1.5				
IL-1RN rs9005	G/G	48	48	30	46.2	25	38.5	0.796	0.973	0.967	0.967
	G/A	40	40	27	41.5	32	49.2				
	A/A	12	12	8	12.3	8	12.3				
IL-1RN rs315943	G/G	3	3	1	1.5	3	4.6	0.586	0.787	0.818	0.787
	G/A	23	23	20	30.8	13	20.0				
	A/A	74	74	44	67.7	49	75.4				
IL-1R1 rs871659	G/G	5	5	1	1.5	1	1.5	0.021	0.014^	0.506	0.031
	G/A	23	23	5	7.7	16	24.6				
	A/A	72	72	59	90.8	48	73.8				
IL-1R1 rs3771202	C/C	5	5	1	1.5	1	1.5	0.112	0.014^	0.506	0.031
	C/G	23	23	5	7.7	16	24.6				
	G/G	72	72	59	90.8	48	73.8				
IL-1R1 rs3917238	C/C	40	40	14	21.5	21	32.3	0.144	0.115	0.598	0.435
	C/T	40	40	31	47.7	30	46.2				
	T/T	20	20	20	30.8	14	21.5				

^Significant with *p* value < 0.017; CS: Chi-square.

**Table 4:** Comparison of IL-1R1 and IL-1RN SNP alleles between groups.

Variable (Allele)		Group						*p* value (CS)	*p* value Control versus Mild	*p* value control versus severe	*p* value mild versus severe
		Control		Mild OA		Moderate severe OA					
		n	%	n	%	n	%				
IL-1R1 rs2287047	G	122	61.0	63	48.5	73	56.2	0.081	0.100	0.447	0.396
	A	78	39.0	67	51.5	57	43.8				
IL-1RN rs419598	T	176	88.0	112	86.2	117	90.0	0.633	0.747	0.747	0.747
	C	24	12.0	18	13.8	13	10.0				
IL-1RN rs9005	G	136	68.0	87	66.9	82	63.1	0.642	0.933	0.904	0.904
	A	64	32.0	43	33.1	48	36.9				
IL-1RN rs315943	G	29	14.5	22	16.9	19	14.6	0.815	1.000	1.000	1.000
	A	171	85.5	108	83.1	111	85.4				
IL-1R1 rs871659	G	33	16.5	7	5.4	18	13.8	0.011	0.013^	0.620	0.053
	A	167	83.5	123	94.6	112	86.2				
IL-1R1 rs3771202	C	33	16.5	7	5.38	18	13.8	0.011	0.002^	0.514	0.02
	G	167	83.5	123	94.6	112	86.2				
IL-1R1 rs3917238	C	120	60	59	45.4	72	55.4	0.033	0.009^	0.406	0.106
	T	80	40	71	54.6	58	44.6				

^Significant with *p* value < 0.017; Chi-square.

We found a higher prevalence of the IL-1R1 rs3917238 genotypes C/T (OR = 1.7, 95% CI: 1.4–2.1) and T/T (OR = 1.9, 95% CI: 1.5–2.5) among those 3 SNPs. A higher prevalence was also found among people aged ≥ 60 years (OR = 2.1, 95% CI: 1.8–2.4) and those with obesity (OR = 3.6, 95% CI: 1.9–6.8). When we performed multivariate analysis by genotype, we found that only older age (≥ 60 years) with an OR of 2.4 (95% CI: 1.3–4.3) and obesity with an OR of 4.4 (95% CI: 1.4–14.1) were associated with primary knee OA, but not for the SNP genotype (Table 5). However, the result was different when stratified by sex. After stratification by sex, those variables remained associated with knee OA only among females, among whom those who were older, had a higher BMI and had SNP rs871659 allele A showed a higher risk of knee OA. We also found that rs3771202 allele G was associated with a higher risk of knee OA among males (Table 6).

**Table 5:** Factors associated with knee OA versus healthy knees by multivariate regression.

Variable		Group (Knee OA vs. Healthy Knees)				*p* value (CS)	OR (95% CI)	*p* value (LR)	AOR (95% CI)
		Healthy knees		Knee OA					
		n	%	n	%				
Sex	Male	28	46.70%	32	53.30%	0.562	Ref		
	Female	72	42.40%	98	57.60%		1.2 (0.9–1.4)		
Age	< 60	71	50.40%	70	49.60%	0.008	Ref	0.002	Ref
	≥ 60*	29	32.60%	60	67.40%		2.1 (1.8–2.4)		2.4 (1.3–4.3)
BMI	Overweight	96	45.90%	113	54.10%	0.018	Ref	0.011	Ref
	Obese*	4	19.00%	17	81.00%		3.6 (1.9–6.8)		4.4 (1.4–14.1)
IL-1R1 rs871659	G/G	5	71.40%	2	28.60%	0.112	Ref		
	G/A	23	52.30%	21	47.70%		2.3 (0.5–10.7)		
	A/A	72	40.20%	107	59.80%		3.7 (0.9–15.3)		
IL-1R1 rs3771202	C/C	5	71.40%	2	28.60%	0.112	Ref		
	C/G	23	52.30%	21	47.70%		2.3 (0.5–10.7)		
	G/G	72	40.20%	107	59.80%		3.7 (0.9–15.3)		
IL-1R1 rs3917238	C/C	40	53.30%	35	46.70%	0.106	Ref		
	C/T	40	39.60%	61	60.40%		1.7 (1.4–2.1)		
	T/T	20	37.00%	34	63.00%		1.9 (1.5–2.5)		

*Significant with *p* value < 0.05; CS: chi-square test; LR: logistic regression.

**Table 6:** Factors associated with knee OA versus healthy knees by multivariate regression.

Variable		Group (Knee OA vs. Healthy Knees)				*p* value (CS)	OR (95% CI)	Knee OA versus Healthy Knees (Males)				*p* value (CS)	OR (95% CI)	Knee OA versus Healthy Knees (Females)				*p* value (CS)	OR (95% CI)
		Healthy Knees		Knee OA				Healthy Knees		Knee OA				Healthy Knees		Knee OA			
		n	%	n	%			n	%	n	%			n	%	n	%		
Sex	Male	28	46.70%	32	53.30%	0.562	Ref	28	46.7%	32	53.3%								
	Female	72	42.40%	98	57.60%		1.2 (0.9–1.4)							72	42.4%	98	57.6%		
Age	< 60	71	50.40%	70	49.60%	0.008*	Ref	15	51.7%	14	48.3%	0.448	Ref	56	50.0%	56	50.0%	0.005*	Ref
	≥ 60*	29	32.60%	60	67.40%		2.1 (1.8–2.4)	13	41.9%	18	58.1%		2.4 (0.5–4.1)	16	27.6%	42	72.4%		2.6 (1.3–5.2)
BMI	Over weight	96	45.90%	113	54.10%	0.018*	Ref	26	48.1%	28	51.9%	0.49	Ref	70	45.2%	85	54.8%	0.017*	Ref
	Obese*	4	19.00%	17	81.00%		3.6 (1.9–6.8)	2	33.3%	4	66.7%		1.8 (0.3–11.0)	2	13.3%	13	86.7%		5.3 (1.2–24.5)
IL-1R1 rs871659	G/G	5	71.40%	2	28.60%	0.112	Ref	2	100.0%	0	0.0%	0.075	Ref	3	60.0%	2	40.0%	0.603	Ref
	G/A	23	52.30%	21	47.70%		2.3 (0.5–10.7)	9	64.3%	5	35.7%		1.8 (0.1–21.5)	14	46.7%	16	53.3%		1.7 (0.2–11.7)
	A/A	72	40.20%	107	59.80%		3.7 (0.9–15.3)	17	38.6%	27	61.4%		6.3 (0.6–62.2)	55	40.7%	80	59.3%		2.2 (0.3–13.5)
IL-1R1 rs3771202	C/C	5	71.40%	2	28.60%	0.112	Ref	2	100.0%	0	0.0%	0.075	Ref	3	60.0%	2	40.0%	0.603	Ref
	C/G	23	52.30%	21	47.70%		2.3 (0.5–10.7)	9	64.3%	5	35.7%		1.8 (0.1–21.5)	14	46.7%	16	53.3%		1.7 (0.2–11.7)
	G/G	72	40.20%	107	59.80%		3.7 (0.9–15.3)	17	38.6%	27	61.4%		6.3 (0.6–62.2)	55	40.7%	80	59.3%		2.2 (0.3–13.5)
IL-1R1 rs3917238	C/C	40	53.30%	35	46.70%	0.106	Ref	12	63.2%	7	36.8%	0.202	Ref	28	50.0%	28	50.0%	0.365	Ref
	C/T	40	39.60%	61	60.40%		1.7 (1.4–2.1)	10	41.7%	14	58.3%		2.4 (0.7–8.2)	30	39.0%	47	61.0%		1.5 (0.8–3.1)
	T/T	20	37.00%	34	63.00%		1.9 (1.5–2.5)	6	35.3%	11	64.7%		3.1 (0.8–12.3)	14	37.8%	23	62.2%		1.6 (0.7–3.8)
rs871659 Allele (n = 460)	A	159	40.4%	235	59.6%	0.001*	2.4 (1.4–4.1)	43	42.2%	59	57.8%	0.022	2.6 (1.2–10.7)	116	39.7%	176	60.3%	0.017*	2.1 (1.1–3.9)
	G	41	62.1%	25	37.9%		Ref	13	72.2%	5	27.8%		Ref	28	58.3%	20	41.7%		Ref
rs3771202 Allele (n = 460)	C	33	56.9%	25	43.1%	0.039	Ref	13	72.2%	5	27.8%	0.022	Ref	20	50.0%	20	50.0%	0.297	Ref
	G	167	41.5%	235	58.5%		1.8 (1.0–3.2)	43	42.2%	59	57.8%		2..6 (1.2–10.7)	124	41.3%	176	58.7%		1.4 (0.7–2.7)
rs3917238 Allele (n = 460)	C	119	47.8%	130	52.2%	0.047	Ref	34	54.8%	28	45.2%	0.06	Ref	85	45.5%	102	54.5%	0.2	Ref
	T	81	38.4%	130	61.6%		1.2 (1.0–1.4)	22	37.9%	36	62.1%		1.9 (0.9–4.1)	59	38.6%	94	61.4%		1.3 (0.8–2.0)

^Significant with *p* value < 0.017.

*Significant with *p* value < 0.05.

We analysed the association between sex, age, BMI, and SNPs of IL-1R1 and IL-1RN and clinical findings. We found an association between moderate-severe VAS scores and obesity with an OR of 2.8 (95% CI: 1.0–7.5). We also found a higher prevalence of moderate-severe VAS scores in IL-1R1_rs3917238 (C/C) (Supplementary Table 1).

We analysed the association between sex, age, BMI, and genetic polymorphisms of IL-1R1 and IL-1RN with EQ-5D-3L. None of the variables were associated with problems in the EQ-5D-3L mobility or anxiety dimension. Regarding the self-care dimension in the EQ-5D-3L, we found that older age was associated with self-care problems, with an OR of 2.3 (95% CI: 1.1–4.9). None of the SNPs were associated with self-care problems (Supplementary Table 2). When we analysed the association of the EQ-5D-3L usual activity dimension with sex, age, BMI, and genetic polymorphisms of IL-1R1 and IL-1RN, the EQ-5D-3L usual activity dimension had an association with older age (OR = 2.3, 95% CI: 1.2–4.5) and obesity (OR = 3.0, 95% CI: 1.1–8.0). None of the SNPs were associated with problems in the EQ-5D-3L usual activity dimension (Supplementary Table 3). Older age (OR = 2.4, 95% CI: 1.4–4.2) and obesity (OR = 3.5, 95% CI: 1.3–9.3) were associated with problems in the EQ-5D-3L pain dimension, while none of the SNPs were associated with pain problems (Supplementary Table 4).

We found an association between radiologic severity and age. Severe OA was associated with older age (≥ 60 years), with an OR of 2.7 (95% CI: 2.1–3.5). We also found a higher prevalence of severe OA in IL-1R1_rs871659 (G/A), IL-1R1_rs3771202 (C/G) and IL-1R1_rs3917238 (C/C); however, after adjustment in multivariate analysis, only older age was associated with severe OA (Supplementary Table 5).

We analysed the association between genetic polymorphisms of IL-1R1 and IL-1RN and IL-1R1 and IL-1Ra protein concentrations in plasma in primary knee OA cases in Indonesia. After stratification by the covariate, we found an association between the gene variant rs871659 genotype A/A, rs3771202 genotype G/G and rs3917238 genotype T/T and the plasma concentration of IL-1R1 protein (Supplementary Table 6). We also found an association between the gene variant rs3917238 genotype T/T and the plasma concentration of IL-1Ra protein (Supplementary Table 7).

We evaluated the association between the haplotypes of the three SNPs and the OA group in this study. Each existing SNP had 2 allele variants, A and G for rs871659, G and C for rs2771202, and T and C for rs3917238. We used a combination of alleles from these three SNPs to perform haplotype analysis. From all samples analysed, only 3 haplotypes from these three SNPs were found, namely, AGT, AGC, and GCC. Major haplotypes were used as a reference to calculate ORs and *p* values. Based on calculations, GCC haplotypes were associated with OA between the control group and the mild group (Supplementary Table 8) but not between the control and severe groups (Supplementary Table 9). The GCC haplotype was also associated with knee OA (Supplementary Table 10). Regarding HWE, we found that in the control versus mild group, rs871659 and rs3771202 did not follow the HWE (*p* value < 0.05) (Supplementary Table 11).

## Discussion

Osteoarthritis is a degenerative disease that is influenced by multiple factors, such as age, BMI, sex, mechanical abnormalities, previous injury, and genetics^11^. Recently, more studies have focused on the relationship between genetic polymorphisms and osteoarthritis. This is the first case–control study to be performed in Indonesia that focuses on the relationship between genetic polymorphisms and knee osteoarthritis and its correlation to clinical findings.

We evaluated the incidence of primary OA, and we compared the healthy knee (control) with the knee OA group (mild, moderate-severe groups) and matched them based on age, sex, and BMI. We still found differences in age and BMI, where patients aged ≥ 60 years had a higher incidence of knee OA than those aged < 60 years. The incidence of primary knee OA was also higher according to BMI, specifically for overweight and obese patients. We also found that age ≥ 60 years was correlated with primary knee OA. Obesity also had a correlation with primary knee OA. These results were also seen in previous studies that showed age and obesity to be associated with knee OA^12–16^. However, we could not find any association between sex and primary knee OA. This finding is different from that of a previous study^12,17^ that found female sex to be a factor associated with knee OA.

In our study, we performed genotype analysis on IL-1RN based on multiple previous studies in East Asia and showed that several polymorphisms were related to primary knee OA and its severity. A study by Attur in 2009 showed the role of IL-1RN variants in radiographic severity. The study revealed that carriers of the CTA haplotype (rs419598/rs315952/rs9005) had a decreased risk for severe knee OA. Another polymorphism at rs419598 was also reported to be associated with radiographic severity^10^. Another study by Wu and colleagues in 2012 showed that an IL-1RN gene variant (rs419598/rs9005/rs315943) was associated with increased progression of knee OA^7^. A study by Ahmed in 2016 showed that genetic variants in IL-1RN (rs9005 and rs315943) were associated with the risk of OA development^8^.

Another study conducted in different geographical areas showed genetic variants of IL-1A (rs1800587), IL-1B (rs1143634), IL-1B (rs16944), and IL-1RN (VNTR). Kaarvatn and his colleagues found a clear association that only affected women. These genetic variants were associated with a sixfold lower risk of knee OA. This finding implicated a novel sex-specific issue to consider regarding OA susceptibility^18^. These findings showed that geographical differences might influence the development of certain diseases and that the precision of ethnicity in sample selection should prevent confounding variables that might interfere with the results of the study.

However, our study showed no association between IL-1RN gene variants rs419598, rs9005, and rs315943 and IL-1R1 rs2287047. Conflicting results were also found in previous systematic reviews and meta-analyses regarding the effect of genetic polymorphisms on the incidence of primary knee OA and clinical, radiologic, and laboratory findings^19^. Studies on the relationship of polymorphisms of the IL-1 family with the severity of knee OA are still scarce.

To provide better gene variant mapping in Indonesian knee OA cases, we expanded primer selection to all variants located in IL-1RN and IL-1R1. This action resulted in different polymorphisms that were related to primary knee OA cases in Indonesia. We found 3 SNPs located in the IL-1R1 gene to be correlated with primary knee OA. These three variants (rs871659, rs3771202, and rs3917238) were found to be associated with predisposing factors for primary knee OA. We found a higher prevalence of knee OA in rs3917238 C/T and T/T. However, when we performed a multivariate analysis with age, sex, and BMI, the impact was diminished. This is probably because the impact of this SNP is less than the impact of age and BMI in primary knee OA. The other reason is probably that this SNP was detected in small amounts; therefore, we need a larger sample size to evaluate whether this SNP impacts the incidence of primary knee OA. However, together with age and BMI, this SNP affected the incidence of primary knee OA. We also found a higher prevalence of severe VAS scores in rs3917238 genotype C/C. We did not find any SNP that correlated with the EQ-5D-3L mobility, EQ-5D-3L self-care, EQ-5D-3L usual activity, EQ-5D-3L pain, or EQ-5D-3L anxiety. We also did not find any genetic factor in the incidence of primary knee OA that correlated with serum levels of IL-1R1 and IL-1Ra.

Osteoarthritis has been considered a genetic disease for the past decade. Some studies have shown a correlation between genetics and osteoarthritis. Almost 100 DNA polymorphisms have been found to correlate with polymorphic DNA^20^. The DNA sequence is copied into an RNA sequence. This messenger RNA (mRNA) is used to produce the protein. A study performed by Shorter showed that miRNA can bind to mRNA and silence gene expression^21^. This results in a decrease or change in the resulting protein. Several studies have shown the important role of miRNA in regulating the homeostasis of joint tissues, especially in OA^22–24^. miRNA itself can suppress the expression of MMP13, IL-6, and IL-8^25^ or can dampen IL-1β expression^26–28^. In this study, we found 3 SNPs that might correlate with OA. However, we did not find any difference in the serum levels of IL-1R1 or IL-1Ra. This finding can be due to several reasons. First, the sample source was serum. Second, miRNA itself can silence the genetic expression of SNPs, which can cause no difference in the protein levels of IL-1R1 and IL-1Ra. Third, the effect of miRNA in causing knee OA is stronger than the effect of the SNP itself. The correlation between miRNA and SNP expression needs to be evaluated.

The three gene variants analysed in our study were located in the intron region of the IL-1R1 gene, with no known function to date, and have not been linked to knee OA in previous studies. A study by Wu in 2016 found a correlation of the rs3771202 gene variant in IL-1R1 with severe preeclampsia^29^, and another study of rs3771202 conducted by Golozar in 2014 showed its correlation to oesophageal squamous cell carcinoma^30^. The other two variants (rs871659 and rs3917238) had never been found to be correlated with any condition. This finding showed the novelty of our study and could lead to further and larger genetic analysis studies in the general population. This finding can also be added to genomic data to be used for further research on knee OA worldwide.

The Hardy–Weinberg equilibrium results showed that these 2 new SNPs did not follow the HWE rule between the control group and the mild OA group. HWE states that genetic variation in a population will always be constant from one generation to the next if there are no external factors (such as mutations, recombination during sexual reproduction, genetic drift, gene migration/gene flow, and natural selection). This does not directly mean that there were external factors that influenced the appearance of these SNPs in the population (patients with mild OA) but may be due to the small number of samples used in this study. The sample size that we used in this study was only 165 (100 controls and 65 individuals with mild OA). To determine whether these two SNPs indeed deviate from the HWE and are not simply due to bias in the data, analysis with a larger sample size is needed.

This study has several limitations. First, in this study, we only focused on a small region of chromosome 2 depicting the IL-1R1 and IL-1RN genes and found four variants that were not correlated but found other 3 variants that were correlated. Only very small regions of the genome can be investigated at a time, meaning that important genes may be overlooked using this method. Second, we only examined IL-1Ra and IL-1R1 from serum and not from synovial fluid. We had difficulty in performing aspiration of the normal knee joint; therefore, we focused on blood examination. One study evaluated the concentration of IL-1β in serum and synovial fluid, and the results showed that the serum level of IL-1β was below the detection limit for RA and OA but not in synovial fluid^31^. Another study evaluated IL-1Ra in serum compared to synovial fluid in the temporomandibular joint. The study showed that the concentration of IL-1Ra in plasma was lower than that in synovial fluid^32^. This may be the reason we could not find any difference in the concentrations of plasma IL-1Ra and IL-1R1 within groups. Third, to examine the genetic effect, a larger sample size is needed. However, in this study with this number of samples, the post hoc power analysis was 84.3%, which showed no type II error in this study. There is no study on the SNP we evaluated and found in Indonesia. Therefore, we cannot evaluate the real homozygote that can be used as a reference. However, even if we decide which homozygote to use as a reference, it will not change the results, only the direction of the correlation. Fourth, we did not elaborate on the role of epigenetics of the interleukin-1 gene. The studies by Genemaras et al.^33^ and Shen et al.^34^ showed the implication of epigenetics in IL-1β and have been implicated in the progression of OA. A separate thorough study of epigenetics in interleukin-1 in the Indonesian population should be performed and analysed together with our results to provide a better understanding of why the gene variant found in this study was not correlated with clinical, radiographic or serum biomarkers.

## Conclusions

In this study, we did not find the correlation between IL-1RN gene variants rs419598, rs9005, and rs315943 and IL-1R1 rs2287047 and knee OA. But we found three SNPs (IL-1R1_rs871659, IL-1R1_rs3771202, and IL-1R1_rs3917238) that might be predisposing factors for primary knee OA. The SNP rs871659 allele A was a predisposing factor for knee OA among females, while the SNP rs3771202 allele G was a predisposing factor for knee OA among males. The evaluation of clinical findings (VAS, KOOS, and EQ-5D-3L), radiographic severity, and laboratory markers of serum IL-1Ra and IL-1R1 protein showed no association with the genetic polymorphisms of the IL-1R1 and IL-1RN genes. Studies on predisposing factors for primary knee OA are still inconclusive. Therefore, further studies with a larger sample size are needed, especially case‒control studies.

## Supplementary Information

Supplementary Tables.

## Data Availability

The data supporting the findings of this study are available from the corresponding author upon reasonable request.
