# Supplementary material for: The role of genetic polymorphisms of interleukin-1 (IL-1R1 and IL-1RN) in primary knee osteoarthritis in Indonesia
Source: Sci Rep. 2023 May 17;13:7967. doi: 10.1038/s41598-023-34824-2 (PMC10192377; doi:10.1038/s41598-023-34824-2)
Supplement: Supplementary file 1 — Supplementary Tables. [file 41598_2023_34824_MOESM1_ESM.docx]

**Supplementary**

Table 1. The association between genetic polymorphism of IL-1R1 and IL-1RN with VAS in primary knee OA case in Indonesia

| Variable | | VAS category | | | | p-value (CS) | OR  (CI 95%) | p-value (LR) | AOR  (CI 95%) |
| --- | --- | --- | --- | --- | --- | --- | --- | --- | --- |
|  |  | **mild** | | **moderate-severe** | |  |  |  |  |
|  |  | **n** | **%** | **n** | **%** |  |  |  |  |
| Sex | Male | 47 | 78.3 | 13 | 21.7 | 0.912 | Ref |  |  |
|  | Female | 132 | 77.6 | 38 | 22.4 |  | 1.0 (0.5-2.1) |  |  |
| Age | <60 | 115 | 81.6 | 26 | 18.4 | 0.086 | Ref |  |  |
|  | >60 | 64 | 71.9 | 25 | 28.1 |  | 1.7 (0.9-3.2) |  |  |
| BMI | Over-  weight | 166 | 79.4 | 43 | 20.6 | 0.065 | Ref | 0.039* | Ref |
|  | Obese | 13 | 61.9 | 8 | 38.1 |  | 2.3(0.9-6.1) |  | 2.8 (1.0-7.5) |
| IL-1R1  rs871659 | G/G | 6 | 85.7 | 1 | 14.3 | 0.216 | Ref |  |  |
|  | G/A | 30 | 68.2 | 14 | 31.8 |  | 2.8 (0.3-25.2) |  |  |
|  | A/A | 143 | 79.9 | 36 | 20.1 |  | 1.5 (0.2-12.9) |  |  |
| IL-1R1  rs3771202 | C/C | 6 | 85.7 | 1 | 14.3 | 0.216 | Ref |  |  |
|  | C/G | 30 | 68.2 | 14 | 31.8 |  | 2.8 (0.3-25.2) |  |  |
|  | G/G | 143 | 79.9 | 36 | 20.1 |  | 1.5 (0.2-12.9) |  |  |
| IL-1R1  rs3917238 | C/C | 52 | 69.3 | 23 | 30.7 | 0.076 | 2.5 (1.0-6.2) | 0.035* | 2.7 (1.1-6.7) |
|  | C/T | 81 | 80.2 | 20 | 19.8 |  | 1.4 (0.6-3.5) | 0.345 | 1.5 (0.6-3.8) |
|  | T/T | 46 | 85.2 | 8 | 14.8 |  | Ref |  | Ref |

Table 2. The association between genetic polymorphism of IL-1R1 and IL-1RN with EQ-5D-3L self-care in primary knee OA case in Indonesia

| Variable | | Self-care | | | | p-value (CS) | | OR  (CI 95%) | p-value (LR) | AOR  (CI 95%) |
| --- | --- | --- | --- | --- | --- | --- | --- | --- | --- | --- |
|  |  | **no problem** | | **some-extreme problem** | | |  |  |  |  |
|  |  | **n** | **%** | **n** | **%** | |  |  |  |  |
| Sex | Male | 53 | 88.3 | 7 | 11.7 | | 0.559 | Ref |  |  |
|  | Female | 145 | 85.3 | 25 | 14.7 | |  | 1.3 (0.5-3.1) |  |  |
| Age | <60 | 127 | 90.1 | 14 | 9.9 | | 0.028* | Ref | 0.031* | Ref |
|  | >60 | 71 | 79.8 | 18 | 20.2 | |  | 2.2 (1.1-4.9) |  | 2.3 (1.1-4.9) |
| BMI | Over-weight | 180 | 86.1 | 29 | 13.9 | | 0.959 | Ref |  |  |
|  | Obese | 18 | 85.7 | 3 | 14.3 | |  | 1.0(0.3-3.7) |  |  |
| IL-1R1  rs871659 | G/G | 6 | 85.7 | 1 | 14.3 | | 0.998 | Ref |  |  |
|  | G/A | 38 | 86.4 | 6 | 13.6 | |  | 0.9 (0.1-9.3) |  |  |
|  | A/A | 154 | 86.0 | 25 | 14.0 | |  | 0.9 (0.1-8.4) |  |  |
| IL-1R1  rs3771202 | C/C | 6 | 85.7 | 1 | 14.3 | | 0.998 | Ref |  |  |
|  | C/G | 38 | 86.4 | 6 | 13.6 | |  | 0.9 (0.1-9.3) |  |  |
|  | G/G | 154 | 86.0 | 25 | 14.0 | |  | 0.9 (0.1-8.4) |  |  |
| IL-1R1  rs3917238 | C/C | 65 | 86.7 | 10 | 13.3 | | 0.934 | Ref |  |  |
|  | C/T | 86 | 85.1 | 15 | 14.9 | |  | 1.1 (0.5-2.7) |  |  |
|  | T/T | 47 | 87.0 | 7 | 13.0 | |  | 0.9 (0.3-2.7) |  |  |

*) significant with p-value <0.05; CS: Chi-square Test; LR: Logistic Regression Multivariate test

Table 3. The association between genetic polymorphism of IL-1R1 and IL-1RN with EQ-5D-3L usual activity in primary knee OA case in Indonesia

| Variable | | Usual activity | | | | p-value (CS) | OR  (CI 95%) | p-value (LR) | AOR  (CI 95%) |
| --- | --- | --- | --- | --- | --- | --- | --- | --- | --- |
|  |  | **no problem** | | **some-extreme problem** | |  |  |  |  |
|  |  | **n** | **%** | **n** | **%** |  |  |  |  |
| Sex | Male | 49 | 81.7 | 11 | 18.3 | 0.513 | Ref |  |  |
|  | Female | 132 | 77.6 | 38 | 22.4 |  | 1.3 (0.6-2.7) |  |  |
| Age | <60 | 118 | 83.7 | 23 | 16.3 | 0.02* | Ref | 0.012* | Ref |
|  | >60 | 63 | 70.8 | 26 | 29.2 |  | 2.1 (1.1-4.0) |  | 2.3 (1.2-4.5) |
| BMI | Over-weight | 168 | 80.4 | 41 | 19.6 | 0.049* | Ref | 0.026* | Ref |
|  | Obese | 13 | 61.9 | 8 | 38.1 |  | 2.5(0.9-6.4) |  | 3.0 (1.1-8.0) |
| IL-1R1  rs871659 | G/G | 6 | 85.7 | 1 | 14.3 | 0.883 | Ref |  |  |
|  | G/A | 35 | 79.5 | 9 | 20.5 |  | 1.5 (0.1-14.5) |  |  |
|  | A/A | 140 | 78.2 | 39 | 21.8 |  | 1.6 (0.2-14.3) |  |  |
| IL-1R1  rs3771202 | C/C | 6 | 85.7 | 1 | 14.3 | 0.883 | Ref |  |  |
|  | C/G | 35 | 79.5 | 9 | 20.5 |  | 1.5 (0.1-14.5) |  |  |
|  | G/G | 140 | 78.2 | 39 | 21.8 |  | 1.6 (0.2-14.3) |  |  |
| IL-1R1  rs3917238 | C/C | 60 | 80.0 | 15 | 20.0 | 0.479 | Ref |  |  |
|  | C/T | 76 | 75.2 | 25 | 24.8 |  | 1.2 (0.5-3.1) |  |  |
|  | T/T | 45 | 83.3 | 9 | 16.7 |  | 1.6 (0.7-3.8) |  |  |

*) significant with p-value <0.05; CS: Chi-square Test; LR: Logistic Regression Multivariate test

Table 4. The association between genetic polymorphism of IL-1R1 and IL-1RN with EQ-5D-3L pain in primary knee OA case in Indonesia

| Variable | | Pain | | | | | p-value (CS) | OR  (CI 95%) | p-value (LR) | AOR  (CI 95%) |
| --- | --- | --- | --- | --- | --- | --- | --- | --- | --- | --- |
|  |  | **no problem** | | **some-extreme problem** | | |  |  |  |  |
|  |  | **n** | **%** | | **n** | **%** |  |  |  |  |
| Sex | Male | 35 | 58.3 | | 25 | 41.7 | 0.802 | Ref |  |  |
|  | Female | 96 | 56.5 | | 74 | 43.5 |  | 1.0 (0.6-1.9) |  |  |
| Age | <60 | 91 | 64.5 | | 50 | 35.5 | 0.02* | Ref | 0.002* | Ref |
|  | >60 | 40 | 44.9 | | 49 | 55.1 |  | 2.2 (1.3-3.8) |  | 2.4 (1.4-4.2) |
| BMI | Over-weight | 124 | 59.3 | | 85 | 40.7 | 0.022* | Ref | 0.011* | Ref |
|  | obese | 7 | 33.3 | | 14 | 66.7 |  | 2.9 (1.1-7.5) |  | 3.5 (1.3-9.3) |
| IL-1R1  rs871659 | G/G | 6 | 85.7 | | 1 | 14.3 | 0.354 | Ref |  |  |
|  | G/A | 24 | 54.5 | | 20 | 45.5 |  | 5.0 (0.5-45.1) |  |  |
|  | A/A | 101 | 56.4 | | 78 | 43.6 |  | 4.6 (0.5-39.3) |  |  |
| IL-1R1  rs3771202 | C/C | 6 | 85.7 | | 1 | 14.3 | 0.354 | Ref |  |  |
|  | C/G | 24 | 54.5 | | 20 | 45.5 |  | 5.0 (0.5-45.1) |  |  |
|  | G/G | 101 | 56.4 | | 78 | 43.6 |  | 4.6 (0.5-39.3) |  |  |
| IL-1R1  rs3917238 | C/C | 46 | 61.3 | | 29 | 38.7 | 0.558 | Ref |  |  |
|  | C/T | 57 | 56.4 | | 44 | 43.6 |  | 1.2 (0.6-2.2) |  |  |
|  | T/T | 28 | 51.9 | | 26 | 48.1 |  | 1.5 (0.7-2.9) |  |  |

*) significant with p-value <0.05; CS: Chi-square Test; LR: Logistic Regression Multivariate test

Table 5. Factors associated to radiographic score primary knee OA (Severe vs Mild OA) with regression multivariate

| Variable | | Group | | | | p-value (CS) | | | OR  (CI 95%) | p-value (LR) | AOR  (CI 95%) |
| --- | --- | --- | --- | --- | --- | --- | --- | --- | --- | --- | --- |
|  |  | **Mild** | | **Severe** | | |  | |  |  |  |
|  |  | **n** | **%** | **n** | **%** | | |  |  |  |  |
| Sex | Male | 18 | 56.3 | 14 | 43.8 | | | 0.415 | Ref |  |  |
|  | Female | 47 | 48.0 | 51 | 52.0 | | |  | 1.4 (1.0-1.9) |  |  |
| Age | <60 | 43 | 61.4 | 27 | 38.6 | | | 0.005 | Ref | 0.008* | Ref |
|  | >60 | 22 | 36.7 | 38 | 63.3 | | |  | 2.7 (2.1-3.6) |  | 2.7 (1.3-5.6) |
| BMI | Over-weight | 58 | 51.3 | 55 | 48.7 | | | 0.435 | Ref |  |  |
|  | obese | 7 | 41.2 | 10 | 58.8 | | |  | 1.5 (0.8-2.6) |  |  |
| IL-1R1  rs871659 | G/G | 59 | 55.1 | 48 | 44.9 | | | 0.032 | Ref |  |  |
|  | G/A | 5 | 23.8 | 16 | 76.2 | | |  | 3.9 (2.1-7.1) |  |  |
|  | A/A | 1 | 50.0 | 1 | 50.0 | | |  | 1.2 (0.1-66.2) |  |  |
| IL-1R1  rs3771202 | C/C | 59 | 55.1 | 48 | 44.9 | | | 0.032 | Ref |  |  |
|  | C/G | 5 | 23.8 | 16 | 76.2 | | |  | 3.9 (2.2-7.1) |  |  |
|  | G/G | 1 | 50.0 | 1 | 50.0 | | |  | 1.2 (0.1-66.2) |  |  |
| IL-1R1  rs3917238 | C/C | 14 | 40.0 | 21 | 60.0 | | | 0.29 | Ref |  |  |
|  | C/T | 31 | 50.8 | 30 | 49.2 | | |  | 0.6 (0.4-0.9) |  |  |
|  | T/T | 20 | 58.8 | 14 | 41.2 | | |  | 0.5 (0.3-0.7) |  |  |

*) significant with p-value <0.05 (CS: Chi-square Test) (LR: Logistic Regression Multivariate test)

Table 6. The association of serum IL-1R1 to Knee OA after adjusted by sex, age, BMI and SNP

| Variables | | | Knee OA vs Healthy Knee | | | | | p-value | Mean Difference (CI 95%) |  |  |
| --- | --- | --- | --- | --- | --- | --- | --- | --- | --- | --- | --- |
|  |  |  | **Healthy Knee** | | **Knee OA** | | |  |  |  |  |
|  |  |  | **Mean** | **SD** | | **Mean** | **SD** |  |  | |  |
| IL-1R1 | Sex | Male | 44.9 | 102.9 | 34.8 | | 96.4 | 0.066 | 10.1 (-41.4 – 61.6) | | |
|  |  | Female | 34.6 | 58.5 | 22.0 | | 32.9 |  | 12.6 (-2.6– 27.7) | | |
|  | Age | <60 | 41.0 | 84.9 | 23.1 | | 33.4 | 0.435 | 17.9 (-3.6 – 39.4) | | |
|  |  | ≥60 | 28.9 | 28.6 | 27.6 | | 73.6 |  | 1.3 (-26.9 – 29.5) | | |
|  | BMI | Over-weight | 37.6 | 74.4 | 26.4 | | 59.4 | 0.281 | 11.2 (-7.1 – 29.4) | | |
|  |  | obese | 34.9 | 41.2 | 17.2 | | 7.8 |  | 17.7 (-100.8 – 136.2) | | |
|  | rs871659 | G/G | 18.7 | 10.3 | 86.7 | | 97.6 | 0.115 | -68 (-163.9 – 27.9) | | |
|  |  | G/A | 27.6 | 25.5 | 43.8 | | 118.7 |  | -16.2 (-67.3 – 34.9) | | |
|  |  | A/A | 42.0 | 84.8 | 20.4 | | 28.9 |  | 21.6 (4.1 – 39.1)* | | |
|  | rs3771202 | C/C | 18.7 | 10.3 | 86.7 | | 97.6 | 0.115 | -68 (-163.9 – 27.9) | | |
|  |  | C/G | 27.6 | 25.5 | 43.8 | | 118.7 |  | -16.2 (-67.3 – 34.9) | | |
|  |  | G/G | 42.0 | 84.8 | 20.4 | | 28.9 |  | 21.6 (4.1 – 39.1)* | | |
|  | rs3917238 | C/C | 29.3 | 26.9 | 23.9 | | 38.0 | 0.378 | 5.4 (-9.6 -20.4) | | |
|  |  | C/T | 43.7 | 101.3 | 27.6 | | 70.3 |  | 16.1 (-17.7 – 49.9) | | |
|  |  | T/T | 41.6 | 71.8 | 22.2 | | 39.3 |  | 19.4 (19.3 – 19.5)* | | |

1. Analysis using Anova two way mean comparison test of IL-1R1 Healthy vs OA Knee after adjusted by each covariate variables
2. Mean differences of IL-1R1 Healthy vs OA Knee analysis stratified by each categorical level of covariate variables; *) significant with CI 95% interval exclude 0 value

Table 7. The association of serum IL-1Ra to Knee OA after adjusted by sex, age, BMI and SNP

| Variables | | | Knee OA vs Healthy Knee | | | | | p-value | Mean Difference (CI 95%) |  |
| --- | --- | --- | --- | --- | --- | --- | --- | --- | --- | --- |
|  |  |  | **Healthy Knee** | | **Knee OA** | | |  |  |  |
|  |  |  | **Mean** | **SD** | | **Mean** | **SD** |  |  |  |
| IL-1Ra | Sex | Male | 515.2 | 260.2 | | 461.8 | 232.2 | 0.135 | 53.4 (-73.8 – 180.6) | |
|  |  | Female | 443.5 | 164.0 | | 489.6 | 183.2 |  | -46.1 (-9.89 – 7.6) | |
|  | Age | <60 | 466.6 | 194.4 | | 487.5 | 196.3 | 0.247 | -20.9 (-85.9 – 44.1) | |
|  |  | ≥60 | 456.2 | 206.5 | | 477.2 | 196.7 |  | -21 (-110.8 – 68.8) | |
|  | BMI | Over-weight | 455.4 | 183.9 | | 478.7 | 197.7 | 0.293 | -23.3 (-75.7 – 29.1) | |
|  |  | obese | 659.5 | 395.2 | | 509.6 | 186.3 |  | 149.9 (-495 – 794) | |
|  | rs871659 | G/G | 436.1 | 99.7 | | 667.8 | 294.5 | 0.896 | -231.7 (-573-110) | |
|  |  | G/A | 432.5 | 216.4 | | 487.0 | 190.6 |  | -54.5 (-179 – 70.1) | |
|  |  | A/A | 475.4 | 196.3 | | 478.4 | 195.7 |  | -3 (-61.9 -55.9) | |
|  | rs3771202 | C/C | 436.1 | 99.7 | | 667.8 | 294.5 | 0.896 | -231.7 (-573-110) | |
|  |  | C/G | 432.5 | 216.4 | | 487.0 | 190.6 |  | -54.5 (-179 – 70.1) | |
|  |  | G/G | 475.4 | 196.3 | | 478.4 | 195.7 |  | -3 (-61.9 -55.9) | |
|  | rs3917238 | C/C | 451.2 | 195.3 | | 504.5 | 201.1 | 0.797 | -53.3 (-144.6 -38.0) | |
|  |  | C/T | 481.5 | 217.2 | | 500.5 | 212.8 |  | -19 (-105.6 – 67.6) | |
|  |  | T/T | 452.4 | 161.1 | | 428.4 | 147.3 |  | 24 (23.9 -24.0)* | |

1. Analysis using Anova two way mean comparison test of IL-1Ra Healthy vs OA Knee after adjusted by each covariate variables
2. Mean differences of IL-1Ra Healthy vs OA Knee analysis stratified by each categorical level of covariate variables; *) significant with CI 95% interval exclude 0 value

Table 8. Haplotype association from 3 SNP between control vs mild group

| Haplotype | | | Freq | OR | 95 % CI | p-value |
| --- | --- | --- | --- | --- | --- | --- |
| rs871659 | rs3771202 | rs3917238 |  |  |  |  |
| A | G | T | 0.4576 | 1.00 | Reference haplotype | - |
| A | G | C | 0.4212 | 0.73 | 0.46 – 1.18 | 0.1975 |
| G | C | C | 0.1212 | 0.32 | 0.14 – 0.71 | 0.0053 |

Table 9. Haplotype association from 3 SNP between control vs severe group

| Haplotype | | | Freq | OR | 95 % CI | p-value |
| --- | --- | --- | --- | --- | --- | --- |
| rs871659 | rs3771202 | rs3917238 |  |  |  |  |
| A | G | C | 0.4273 | 1.00 | Reference haplotype | - |
| A | G | T | 0.4182 | 1.15 | 0.72 – 1.81 | 0.5604 |
| G | C | C | 0.1545 | 0.89 | 0.47 – 1.70 | 0.7283 |

Table 10. Haplotype association from 3 SNP between healthy knee vs knee OA

| Haplotype | | | Freq | OR | 95 % CI | p-value |
| --- | --- | --- | --- | --- | --- | --- |
| rs871659 | rs3771202 | rs3917238 |  |  |  |  |
| A | G | T | 0.4543 | 1.00 | Reference haplotype | - |
| A | G | C | 0.4196 | 0.79 | 0.53 – 1.16 | 0.2244 |
| G | C | C | 0.1261 | 0.52 | 0.30 – 0.91 | 0.0218 |

Table 11. HWE p.value - Test the null hypothesis that Hardy-Weinberg equilibrium holds in cases, controls and both populations

| SNP | HWE p.value | | |
| --- | --- | --- | --- |
|  | Control vs OA | Control vs Mild | Control vs Severe |
| rs871659 | 0.064 | 0.0169* | 0.2301 |
| rs3771202 | 0.064 | 0.0169* | 0.2301 |
| rs3917238 | 0.0846 | 0.0864 | 0.1097 |
